# Potential anatomic risk factors resulting oversized postoperative medial proximal tibial angle after double level osteotomy

**DOI:** 10.1186/s12891-022-06101-2

**Published:** 2022-12-23

**Authors:** Shuntaro Nejima, Ken Kumagai, Shunsuke Yamada, Masaichi Sotozawa, Dan Kumagai, Hironori Yamane, Yutaka Inaba

**Affiliations:** Department of Orthopaedic Surgery, Yokohama City University School of Medicine, 3-9 Fukuura, Kanazawa-Ku, Yokohama, 236-0004 Japan

**Keywords:** Open wedge high tibial osteotomy, Closed wedge high tibial osteotomy, Double level osteotomy, Distal femoral osteotomy, Osteotomies around the knee

## Abstract

**Background:**

Double level osteotomy (DLO) has been introduced to prevent increased postoperative joint line obliquity. However, although DLO is planned, knees with postoperative medial proximal tibial angle (MPTA) > 95° in preoperative surgical planning are present. This retrospective study aimed to evaluate risk factors for an MPTA > 95° in preoperative surgical planning for DLO in patients with varus knee osteoarthritis (OA).

**Methods:**

A total of 168 knees that underwent osteotomies around the knee for varus knee OA were enrolled. The hip-knee-ankle angle (HKA), weight-bearing line (WBL) ratio, mechanical lateral distal femoral angle (mLDFA), joint line convergence angle (JLCA) and mechanical medial proximal tibial angle (mMPTA) were measured on preoperative radiographs. The postoperative WBL ratio was planned to be 62.5%. When the postoperative mMPTA was more than 95° in isolated high tibial osteotomy (HTO), (DLO) was planned so that the postoperative mLDFA was 85°, and residual deformity was corrected by HTO. Knees with postoperative mMPTA ≤ 95° and > 95° were classified into the correctable group and uncorrectable group, respectively.

**Results:**

DLO was required in 101 knees (60.1%). Among them, 41 knees (40.6%) were classified into the uncorrectable group. Binomial logistic regression analysis showed that preoperative JLCA and mMPTA were independent predictors in the uncorrectable group.

**Conclusions:**

Even with DLO, postoperative mMPTA was more than 95° in approximately 40% of cases. Preoperative increased JLCA and decreased mMPTA were risk factors for a postoperative mMPTA of > 95° after DLO.

## Background

High tibial osteotomy (HTO) is a well-established surgical treatment for medial knee osteoarthritis (OA) with varus malalignment [1–3]. Meanwhile, in isolated HTO, excessive postoperative overcorrection of a medial proximal tibial angle (MPTA) of > 95° leads to increased shear stress on the articular cartilage [4] and consequent inferior clinical outcomes [5, 6]. To maintain an ideal MPTA, double-level osteotomy (DLO) was introduced, and good clinical outcomes were reported [7–12]. Feucht et al. [13] reported that 33% of patients with varus malalignment required DLO to avoid postoperative MPTA > 95° in surgical planning. However, although DLO is planned, we sometimes encounter knees with postoperative MPTA > 95° in preoperative surgical planning. Theoretically, an intraarticular deformity affects the postoperative bone morphology in surgical planning because the intraarticular deformity cannot be corrected by osteotomies around the knee. Thus, the purpose of this retrospective study was to evaluate risk factors for postoperative MPTA > 95° in preoperative surgical planning for DLO in patients with medial knee OA with varus alignment. It was hypothesized that a preoperative increased joint line convergence angle (JLCA) was a risk factor for a postoperative MPTA of > 95° in knees required for DLO.

## Methods

The inclusion criteria of osteotomies around the knee were based on our surgical indication: medial knee OA with varus alignment. Exclusion criteria were symptomatic lateral compartmental or patellofemoral OA, a history of joint infection or inflammatory arthritis, and flexion contracture > 15°. Thus, a total of 156 patients (195 knees) who underwent osteotomies around the knee for medial knee OA with varus alignment from June 2016 to July 2021 were enrolled in this cross-sectional study. Patients with a history of surgical treatment of the lower limbs (10 knees, meniscus repair or resection; 5 knees, fixation of a lower limb fracture; 4 knees, anterior cruciate ligament reconstruction; 1 knee, total hip arthroplasty) or a concomitant procedure (5 knees, tibial tubercle transfer; 2 knees, anterior cruciate ligament reconstruction) were excluded. Thus, 136 patients (168 knees) met the inclusion criteria for this study.

### Measurements of radiological parameters

For preoperative surgical planning, anteroposterior whole-leg radiographs were obtained in the one-leg standing position, with the knee in full extension. The X-ray beam was centred on the knee with the patella facing forwards. The hip-knee-ankle angle (HKA), weight-bearing line (WBL) ratio, mechanical lateral distal femoral angle (mLDFA), JLCA and mechanical medial proximal tibial angle (mMPTA) were measured. The measurements of radiological parameters and surgical planning were performed using Fujifilm OP-A software (Fujifilm Co. Ltd., Tokyo, Japan). The HKA was defined as the angle between the mechanical axes of the femur and tibia, with a positive value representing valgus alignment. The WBL ratio was defined as the ratio of the distance from the medial edge of the tibial plateau to the intersection of the weight-bearing line to the length of the tibial plateau. The WBL ratio reflected the medial tibial edge at 0% and the lateral tibial edge at 100%. The mLDFA was defined as the lateral angle between the mechanical axis of the femur and the tangent of the femoral condyles. The JLCA was defined as the angle between the tangent of the femoral condyles and the joint line of the proximal tibia. A JLCA with an acute angle at the medial side was defined as positive. The mMPTA was defined as the medial angle between the mechanical axis of the tibia and the joint line of the tibia. Good intra- and interobserver reliability has been reported for these measurements [13].

### Surgical planning of osteotomies around the knee

**Fig. 1:**
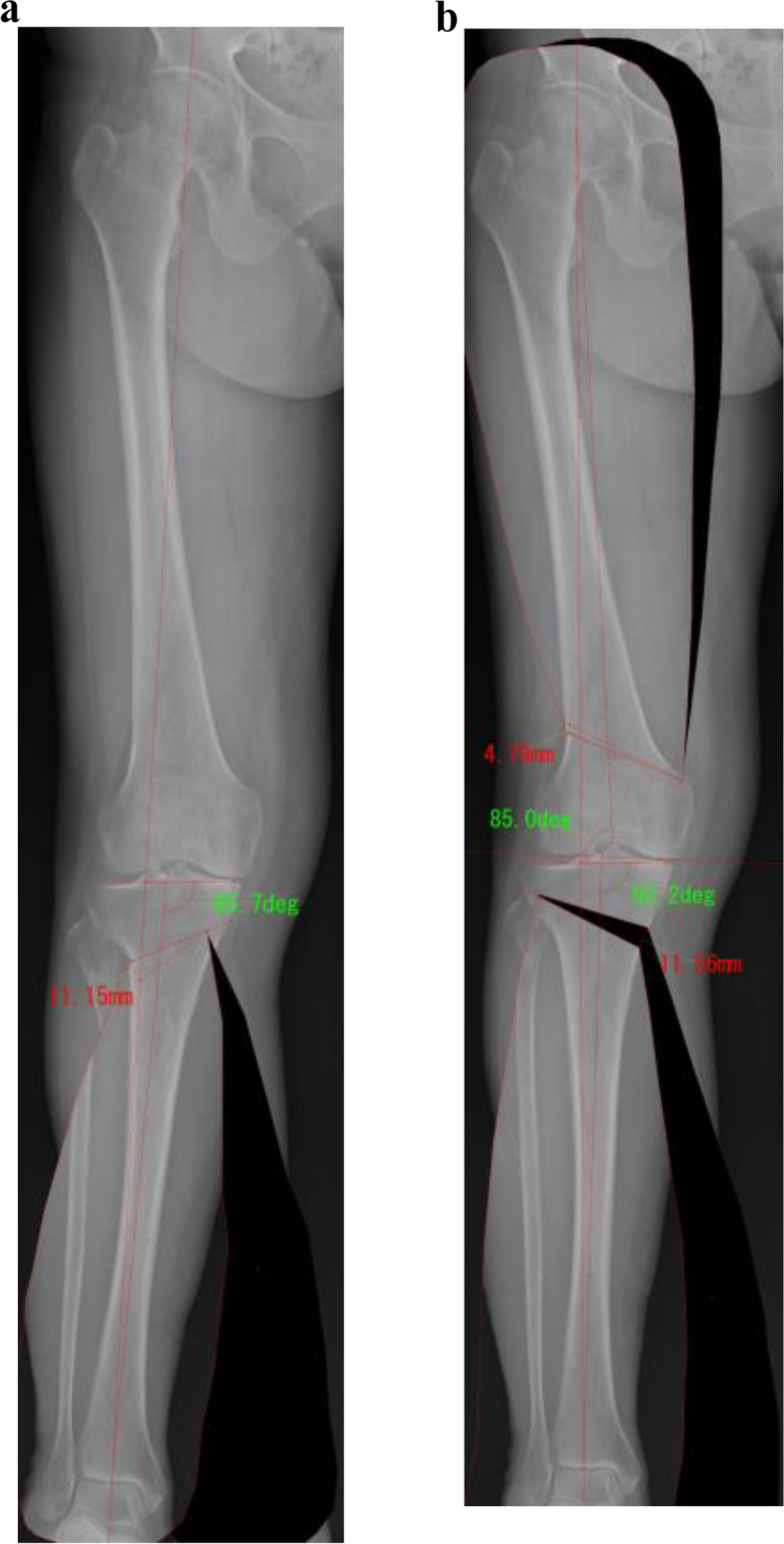
In this case, the postoperative mechanical medial proximal tibial angle (mMPTA) was 95.7° in closed wedge high tibial osteotomy (HTO) (**a**). When the postoperative mMPTA is more than 95° in isolated HTO, double-level osteotomy (DLO) is planned. In DLO, lateral closed wedge distal femoral osteotomy is planned so that the postoperative mechanical lateral distal femoral angle is 85°. Then, the residual deformity is corrected with HTO. This case was classified into the correctable group because the postoperative mMPTA was ≤ 95° (**b**)

Surgical planning was performed so that the postoperative WBL ratio was 62.5%. First, open wedge high tibial osteotomy (OWHTO) was planned. The transverse cut line was located 35 mm below the medial tibial plateau to the safe zone [14]. The lateral hinge was set 5 mm medial to the lateral cortex of the tibia. The transverse cut line was opened to accomplish the target point of the postoperative alignment. If the opening width was more than 13 mm, closed wedge HTO (CWHTO) was planned alternatively because an opening width over 13 mm was a risk factor for delayed bone healing and progression of patellofemoral cartilage injuries [15, 16]. CWHTO was planned in hybrid style [17–19]. A transverse cut was planned 40 mm below the lateral tibial plateau to the inflection point of the medial tibial cortex. The hinge point was set to divide the transverse cut line by 3 to 1. When the postoperative mMPTA was more than 95° in isolated HTO, DLO was planned (Fig. 1a) [10, 11]. In DLO, lateral closed-wedge distal femoral osteotomy (DFO) was planned so that the postoperative mLDFA was 85° [10, 20]. A transverse cut was planned 40 mm above the lateral epicondyle of the femur. The hinge point was set between the medial condyle and cortex of the femur. Then, residual deformity was corrected with HTO. After planning, in DLO group, knees with postoperative mMPTA ≤ 95° and > 95° were classified into the correctable group and uncorrectable group, respectively (Fig. 1b). This study was approved by the institutional review board of Yokohama City University (F211100004), and written informed consent was obtained from each patient.

### Statistical analysis

Data are expressed as means ± standard deviation. In knees with required for DLO, demographic data and preoperative radiological parameters were compared between correctable and uncorrectable groups using unpaired t tests and chi-squared tests. Binomial logistic regression analysis with the forwards selection method was performed to identify risk factors for the uncorrectable group in knees required for DLO. All statistical analyses were performed using IBM SPSS for Windows, version 27.0 (IBM Corporation, Armonk, NY, USA). *P* values less than 0.05 were considered significant. A power analysis was performed on comparisons using an unpaired t test (significance level = 0.05, effect size = 0.5) using G*Power version 3.1.9.2 (Heinrich-Heine-Universität, Düsseldorf, Germany). A post hoc power analysis resulted in a power of 0.69.

## Results

The patient characteristics are shown in Table 1. Of the 168 knees, DLO was indicated in 101 knees (60.1%) (Table 2). Of the 101 knees required for DLO, 41 knees (40.6%) were classified into the uncorrectable group. The preoperative demographic and radiological data in knees required for DLO are shown in Table 3. Compared to the correctable group, the preoperative HKA, WBL ratio, and JLCA in the uncorrectable group were more varus and mLDFA more valgus. Binomial logistic regression analysis with the forwards selection method showed that gender, preoperative JLCA and mMPTA were included and that age, body mass index, KL grade, preoperative HKA, WBL ratio, and mLDFA were excluded. Preoperative JLCA and mMPTA were independent predictors in the uncorrectable group in knees required for DLO (Table 4).

**Table 1:** Patients’ demographic characteristics and radiographic data

Knees, n	168
Age, y	65.7 ± 8.4 (45 – 84)
Height, cm	159.2 ± 9.8 (140.1 – 194.3)
Weight, kg	67.2 ± 12.6 (45.3 – 111.7)
Body mass index, kg/m^2^	26.4 ± 3.7 (19.3 – 40.2)
Side, left/right	77/91
Gender, male/female	58/110
Kellgren-Laurence grade 1/2/3/4	0/8/39/121
HKA, °	− 8.9 ± 3.8 (− 20.8 – − 0.1)
WBL ratio, %	6.8 ± 17.0 (− 38.7 – 44.0)
mLDFA, °	87.6 ± 2.1 (81.9 – 93.6)
JLCA, °	5.3 ± 2.3 (0.2 – 12.5)
mMPTA, °	84.1 ± 2.5 (75.4 – 90.1)

Data are presented as means ± standard deviation with the range in parentheses

**Table 2:** The types of osteotomies

		Knees, n	Total
HTO	OWHTO	49	67 (39.9%)
	CWHTO	18	
DFO	DFO	0	0 (0%)
DLO	DFO + OWHTO	63	101 (60.1%)
	DFO + CWHTO	38	
Total		168	168

*HTO* high tibial osteotomy, *OWHTO* open wedge high tibial osteotomy, *CWHTO* closed wedge high tibial osteotomy, *DFO* distal femoral osteotomy, *DLO* double level osteotomy

**Table 3:** Preoperative demographic and radiographic data in knees required for double level osteotomy

	Correctable (*n* = 60)	Uncorrectable (*n* = 41)	*P* value
Age, y	66.8 ± 8.6 (45 – 84)	65.2 ± 9.0 (48 – 83)	n.s
Height, cm	158.1 ± 8.5 (143.8 – 176.9)	158.2 ± 9.4 (142.0 – 179.0)	n.s
Weight, kg	66.6 ± 11.4 (45.3 – 90.2)	69.2 ± 16.5 (47.7 – 111.7)	n.s
Body mass index, kg/m^2^	26.6 ± 4.0 (20.0 – 40.2)	27.3 ± 4.1 (20.5 – 35.8)	n.s
Side, left/right	27/33	20/21	n.s
Gender, male/female	21/39	12/29	n.s
Kellgren-Laurence grade 1/2/3/4	0/1/7/52	0/0/2/39	n.s
HKA, °	− 9.8 ± 3.2 (− 20.8 – − 3.5)	− 12.2 ± 2.7 (− 19.4 – − 6.6)	< 0.001
WBL ratio, %	4.1 ± 13.6 (− 36.9 – 32.6)	− 9.1 ± 12.8 (− 38.7 – 15.0)	< 0.001
mLDFA, °	89.1 ± 1.7 (86.5 – 93.6)	87.9 ± 1.4 (85.7 – 91.9)	< 0.001
JLCA, °	5.0 ± 1.2 (2.2 – 7.2)	8.2 ± 1.4 (6.3 – 12.5)	< 0.001
mMPTA, °	84.3 ± 2.9 (75.4 – 90.1)	83.8 ± 2.4 (79.1 – 88.4)	n.s

Data are presented as means ± standard deviation with the range in parentheses

*HKA* hip-knee-ankle angle, *WBL* weight bearing line, *mLDFA* mechanical lateral distal femoral angle, *JLCA* joint line convergence angle, *mMPTA* mechanical medial proximal tibial angle

**Table 4:** Binomial logistic regression analysis of risk factors for uncorrectable group

Variables	Coefficient	Standard error	Wald	Odds ratio (95% CI)	*P* value
Gender	− 3.134	1.696	3.413	0.044 (0.002 – 1.21)	n.s
JLCA	7.718	2.711	8.106	2247.468 (11.076 – 456,045.094)	< 0.01
mMPTA	− 0.761	0.311	5.999	0.467 (0.254 – 0.859)	< 0.05

*JLCA* joint line convergence angle, *mMPTA* mechanical medial proximal tibial angle

## Discussion

The most important finding of this study was that even with DLO, postoperative mMPTA was more than 95° in 40.6% of cases. In addition, a preoperatively increased JLCA and decreased mMPTA were risk factors for a postoperative mMPTA of > 95° in the surgical planning of DLO. Fuecht et al. [13] reported that when the upper limit of postoperative mMPTA was 95°, DLO was required in 33% of cases, and that anatomic join orientation angle was not obtained in 2% of all cases. Both of these proportions appear to be lower than those in the present study. This may be because the postoperative target alignment in the present study is more valgus than that in their study, and because JLCA of the patients in the present study is larger than that in their study, requiring more correction at the femur and tibia. Although the subjects of their study included knees with KL grades of 0–4, and clinical symptoms were not taken into consideration, the present study included only medial knee OA with varus alignment undergoing osteotomies around the knee. This study demonstrated that in patients requiring osteotomies around the knee, DLO is required in approximately 60% of cases, and even if DLO is performed, postoperative mMPTA exceeds 95° in approximately 40% of cases.

A preoperatively lower HKA was associated with nonanatomical correction for tibial abnormalities after osteotomies around the knee in a previous study [21]. However, the HKA consists of deformities in the mLDFA, JLCA and mMPTA. The present study showed that a preoperatively increased JLCA was a risk factor in the uncorrectable group after DLO. This is reasonable because an intraarticular deformity cannot be corrected by osteotomy. On the other hand, a JLCA decreases after surgery and it leads to overcorrection of the whole-leg alignment in osteotomies around the knee [22–25]. In a previous study, preoperative JLCA ≥ 4° is one of the risk factors for overcorrection due to the change in JLCA [25]. To predict the postoperative JLCA, some techniques, such as lateral-wedge insole or preoperative supine radiographs, are useful [26–28]. Although it is difficult to predict postoperative JLCA changes accurately, these techniques seem to be effective not only to avoid postoperative overcorrection of the whole-leg alignment but also to avoid oversized postoperative mMPTA after osteotomies around the knee.

Preoperative decreased mMPTA was also a risk factor in the uncorrectable group in this study. Although this reason is unclear, it may be that in cases with preoperative decreased mMPTA, CWHTO tends to be necessary because OWHTO results in a larger opening width. In the same postoperative WBL ratio, the postoperative mMPTA could be larger after CWHTO than OWHTO [29] because the leg length after CWHTO was shorter than that after OWHTO [30]. Meanwhile, the adverse effect of the postoperative MPTA on clinical outcomes has been reported only for OWHTO [5, 6]. Goto et al. [31] found that postoperatively increased JLCA, not MPTA, adversely affected long-term clinical outcomes in CWHTO. The relationship between pre- and postoperative joint orientation angles on the coronal plane and clinical outcomes in various osteotomies should be evaluated in the future.

There were a few limitations in this study. First, there are various cut-off values of normal joint orientation angles, target points of postoperative alignment and algorithms for DLO [8–13, 21]. In the present study, the target point of the postoperative WBL ratio was 62.5%, the lower limit of mLDFA was 85°, and the upper limit of mMPTA was 95°. In DLO, lateral closed DFO was planned so that the postoperative mLDFA was 85° and residual deformity was corrected by HTO. It is not clear which method is best. Second, this study focused on radiological surgical planning without clinical outcomes in patients who underwent osteotomies around the knee. Third, the statistical power of this study was slightly weak.

In daily clinical practice, surgeons should be aware that DLO is required in more than half of osteotomies for osteoarthritic knees with varus alignment, and that even with DLO, oversized postoperative mMPTA exists in approximately 40% of cases. If the preoperative JLCA is large in such cases, the use of techniques such as lateral-wedge insole or preoperative supine radiographs to predict the postoperative JLCA should be considered.

## Conclusions

Even with DLO, postoperative mMPTA was more than 95° in approximately 40% of cases. A preoperatively increased JLCA and decreased mMPTA were risk factors for postoperative mMPTA > 95° after DLO.

## Data Availability

The datasets used and/or analysed during the current study available from the corresponding author on reasonable request.

## Abbreviations

HTO: High tibial osteotomy
OA: Osteoarthritis
MPTA: Medial proximal tibial angle
DLO: Double level osteotomy
WBL: Weight-bearing line
JLCA: Joint line convergence angle
HKA: Hip-knee-ankle angle
mLDFA: Mechanical lateral distal femoral angle
mMPTA: Mechanical medial proximal tibial angle
OWHTO: Open wedge high tibial osteotomy
CWHTO: Closed wedge high tibial osteotomy
DFO: Distal femoral osteotomy
KLgrade: Kellgren-Laurence grade

## References

[1] Lobenhoffer P, Agneskirchner JD (2003). Improvements in surgical technique of valgus high tibial osteotomy. Knee Surg Sports Traumatol Arthrosc.

[2] Staubli AE, Simoni CD, Babst R, Lobenhoffer P (2003). TomoFix: a new LCP-concept for open wedge osteotomy of the medial proximal tibia–early results in 92 cases. Injury.

[3] Saito T, Kumagai K, Akamatsu Y, Kobayashi H, Kusayama Y (2014). Five-to ten-year outcome following medial opening-wedge high tibial osteotomy with rigid plate fixation in combination with an artificial bone substitute. J Bone Joint Surg.

[4] Nakayama H, Schröter S, Yamamoto C, Iseki T, Kanto R, Kurosaka K, Kambara S, Yoshiya S, Higa M (2018). Large correction in opening wedge high tibial osteotomy with resultant joint-line obliquity induces excessive shear stress on the articular cartilage. Knee Surg Sports Traumatol Arthrosc.

[5] Akamatsu Y, Kumagai K, Kobayashi H, Tsuji M, Saito T (2018). Effect of increased coronal inclination of the tibial plateau after opening-wedge high tibial osteotomy. Arthroscopy.

[6] Schuster P, Geßlein M, Schlumberger M, Mayer P, Mayr R, Oremek D, Frank S, Schulz-Jahrsdörfer M, Richter J (2018). Ten-year results of medial open-wedge high tibial osteotomy and chondral resurfacing in severe medial osteoarthritis and varus malalignment. Am J Sports Med.

[7] Babis GC, An KN, Chao  EY, Rand  JA, Sim  FH (2002). Double level osteotomy of the knee: a method to retain joint-line obliquity. clinical results. J Bone Joint Surg Am.

[8] Saragaglia D, Blaysat M, Mercier N, Grimaldi M (2012). Results of forty two computer-assisted double level osteotomies for severe genu varum deformity. Int Orthop.

[9] Schröter S, Nakayama H, Yoshiya S, Stöckle U, Ateschrang A, Gruhn J (2019). Development of the double level osteotomy in severe varus osteoarthritis showed good outcome by preventing oblique joint line. Arch Orthop Trauma Surg.

[10] Nakayama H, Iseki T, Kanto R, Kambara S, Kanto M, Yoshiya S, Schröter S (2020). Physiologic knee joint alignment and orientation can be restored by the minimally invasive double level osteotomy for osteoarthritic knees with severe varus deformity. Knee Surg Sports Traumatol Arthrosc.

[11] Akamatsu Y, Nejima S, Tsuji M, Kobayashi H, Muramatsu S. Joint line obliquity was maintained after double-level osteotomy, but was increased after open-wedge high tibial osteotomy. Knee Surg Sports Traumatol Arthrosc 2021;10.1007/s00167-020-06430-6 10.1007/s00167-020-06430-6 33433634

[12] Grasso F, Martz P, Micicoi G, Khakha R, Kley K, Hanak L, Ollivier M, Jacquet C. Double level knee osteotomy using patient-specific cutting guides is accurate and provides satisfactory clinical results: a prospective analysis of a cohort of twenty-two continuous patients. Int Orthop 2021;10.1007/s00264-021-05194-z 10.1007/s00264-021-05194-z 34536082

[13] Feucht MJ, Winkler PW, Mehl J, Bode G, Forkel P, Imhoff AB, Lutz PM (2021). Isolated high tibial osteotomy is appropriate in less than two-thirds of varus knees if excessive overcorrection of the medial proximal tibial angle should be avoided. Knee Surg Sports Traumatol Arthrosc.

[14] Han SB, Lee DH, Shetty GM, Chae DJ, Song JG, Nha KW (2013). A "safe zone" in medial open-wedge high tibia osteotomy to prevent lateral cortex fracture. Knee Surg Sports Traumatol Arthrosc.

[15] Goshima K, Sawaguchi T, Shigemoto K, Iwai S, Nakanishi A, Inoue D, Shima Y (2019). Large opening gaps, unstable hinge fractures, and osteotomy line below the safe zone cause delayed bone healing after open-wedge high tibial osteotomy. Knee Surg Sports Traumatol Arthrosc.

[16] Tanaka T, Matsushita T, Miyaji N, Ibaraki K, Nishida K, Oka S, Araki D, Kanzaki N, Hoshino Y, Matsumoto T, Kuroda R (2019). Deterioration of patellofemoral cartilage status after medial open-wedge high tibial osteotomy. Knee Surg Sports Traumatol Arthrosc.

[17] Takeuchi R, Ishikawa H, Miyasaka Y, Sasaki Y, Kuniya T, Tsukahara S (2014). A novel closed-wedge high tibial osteotomy procedure to treat osteoarthritis of the knee: hybrid technique and rehabilitation measures. Arthrosc Tech.

[18] Ishimatsu T, Takeuchi R, Ishikawa H, Yamaguchi Y, Maeyama A, Osawa K, Jung WH (2019). Hybrid closed wedge high tibial osteotomy improves patellofemoral joint congruity compared with open wedge high tibial osteotomy. Knee Surg Sports Traumatol Arthrosc.

[19] Otsuki S, Murakami T, Okamoto Y, Nakagawa K, Okuno N, Wakama H, Neo M (2019). Hybrid high tibial osteotomy is superior to medial opening high tibial osteotomy for the treatment of varus knee with patellofemoral osteoarthritis. Knee Surg Sports Traumatol Arthrosc.

[20] Paley D, Herzenberg JE, Tetsworth K, McKie J, Bhave A (1994). Deformity planning for frontal and sagittal plane corrective osteotomies. Orthop Clin North Am.

[21] Micicoi G, Grasso F, Kley K, Favreau H, Khakha R, Ehlinger M, Jacquet C, Ollivier M (2021). Osteotomy around the knee is planned toward an anatomical bone correction in less than half of patients. Orthop Traumatol Surg Res.

[22] Lee DH, Park SC, Park HJ, Han SB (2016). Effect of soft tissue laxity of the knee joint on limb alignment correction in open-wedge high tibial osteotomy. Knee Surg Sports Traumatol Arthrosc.

[23] Ogawa H, Matsumoto K, Ogawa T, Takeuchi K, Akiyama H (2016). Preoperative varus laxity correlates with overcorrection in medial opening wedge high tibial osteotomy. Arch Orthop Trauma Surg.

[24] Lee DK, Wang JH, Won Y, Min YK, Jaiswal S, Lee BH, Kim JY (2020). Preoperative latent medial laxity and correction angle are crucial factors for overcorrection in medial open-wedge high tibial osteotomy. Knee Surg Sports Traumatol Arthrosc.

[25] Park JG, Kim JM, Lee BS, Lee SM, Kwon OJ, Bin SI (2020). Increased preoperative medial and lateral laxity is a predictor of overcorrection in open wedge high tibial osteotomy. Knee Surg Sports Traumatol Arthrosc.

[26] Akasaki Y, Mizu-Uchi H, Hamai S, Tsushima H, Kawahara S, Horikawa T, Nakashima Y (2020). Patient-specific prediction of joint line convergence angle after high tibial osteotomy using a whole-leg radiograph standing on lateral-wedge insole. Knee Surg Sports Traumatol Arthrosc.

[27] Shin KH, Jung JK, Nam JJ, Jang KM, Han SB (2020). Preoperative supine radiographs are more accurate than standing radiographs for preoperative planning in medial open-wedge high tibial osteotomy. Arthroscopy.

[28] Akamatsu Y, Nejima S, Tsuji M, Kobayashi H, Muramatsu S (2021). Open-wedge high tibial osteotomy using intraoperative control of joint line convergence angle with reference to preoperative supine radiograph. Arch Orthop Trauma Surg.

[29] Ogino T, Kumagai K, Yamada S, Akamatsu T, Nejima S, Sotozawa M, Inaba Y (2020). Relationship between the bony correction angle and mechanical axis change and their differences between closed and open wedge high tibial osteotomy. BMC Musculoskelet Disord.

[30] Kim JH, Kim HJ, Lee DH (2017). Leg length change after opening wedge and closing wedge high tibial osteotomy: a meta-analysis. PLoS ONE.

[31] Goto N, Akasaki Y, Okazaki K, Kuwashima U, Iwasaki K, Kawamura H, Mizu-uchi H, Hamai S, Tsushima H, Kawahara S, Nakashima Y (2020). The influence of post-operative knee coronal alignment parameters on long-term patient-reported outcomes after closed-wedge high tibial osteotomy. J Orthop.

